# Crystal structure of *catena-*poly[calcium-di-μ_3_-benzoato-κ^6^ *O*,*O*′:*O*-μ_2_-(dimethyl sulfoxide)-κ^2^ *O*:*O*]

**DOI:** 10.1107/S2056989015012487

**Published:** 2015-07-08

**Authors:** Anna S. Voronova, Svitlana R. Petrusenko, Evgeny Goreshnik

**Affiliations:** a Department of Inorganic Chemistry, Taras Shevchenko National University of Kyiv, Volodymyrska str. 64/13, 01601 Kyiv, Ukraine; b Department of Inorganic Chemistry and Technology, Jožef Stefan Institute, Jamova 39, 1000 Ljubljana, Slovenia

**Keywords:** crystal structure, calcium benzoate, coordination polymer, C—H⋯π inter­actions

## Abstract

In the title complex, [Ca(C_7_H_5_O_2_)_2_(C_2_H_6_OS)]_*n*_, the Ca^2+^ ion (site symmetry *m*..) is surrounded by eight O atoms, six from two bridging–chelating tridentate benzoate carboxyl groups and two from a bridging dimethyl sulfoxide mol­ecule (point group symmetry *m*..), giving an irregular coordination geometry [Ca—O bond length range = 2.345 (2)–2.524 (2) Å]. One-dimensional coordination complex chains extending parallel to *c* are generated in which the triply μ_2_-O-bridged Ca^2+^ cations are separated by 3.6401 (5) Å. In the crystal, weak intra­chain C—H⋯π hydrogen bonds are present between the methyl H atoms of the dimethyl sulfoxide mol­ecules as donors and the aromatic rings as acceptors [C—H⋯*Cg* = 3.790 (4) Å].

## Abstract 2

A novel calcium benzoate complex, [Ca(C_7_H_5_O_2_)_2_(C_2_H_6_OS)], has been synthesized and structurally characterized. The compound has a chain polymeric structure stabilized by C—H⋯π inter­actions.

## Chemical context   

Compounds of benzoic acid with calcium are of special inter­est due to their wide-ranging applications, for example as a preservative in the food industry, in cosmetics and in medicine. In spite of that, the crystal structures of such compounds have been poorly investigated. Searches of the Cambridge Structural Database (CSD; Version 5.35, November 2013 + 2 updates; Groom & Allen, 2014 ▸) for simple calcium benzoate complexes revealed only three results: [Ca(benz)_2_(dmf)(H_2_O)]_*n*_ (Yano *et al.*, 2001 ▸), {[Ca(benz)(H_2_O)_3_]^+^ (benz)^−^}_*n*_ (Senkovska & Thewalt, 2005 ▸) and [Ca(benz)_2_(Hbenz)(H_2_O)]_*n*_ (Azizov *et al.*, 2011 ▸) (where benz = benzoate). 
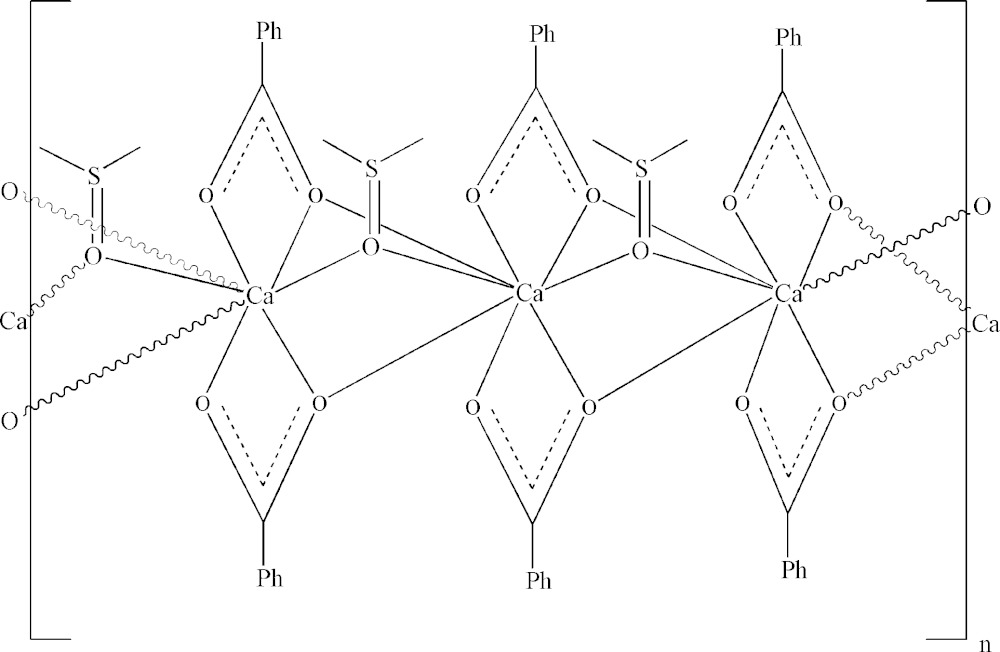


Here we report the synthesis of a new calcium benzoate–dimethyl sulfoxide complex, [Ca(benz)_2_(dmso)]_*n*_, which was obtained as a by-product of an attempted synthesis of an Mn/Cu heterometallic complex (in crystalline form available for X-ray analysis) from the system: Mn–Cu–(bhz–sal)–CaO–KSCN–dmso (in open air), where manganese and copper were used as unactivated metal powders, bhz = benzohydrazide and sal = salicyl­aldehyde. The investigation of the system was carried out as a part of systematic research on the elaboration the ‘direct synthesis’ approach to both homo- and heterometallic coordination compounds (Babich *et al.*, 1996 ▸; Buvaylo *et al.*, 2005 ▸; Vassilyeva *et al.*, 1997 ▸). It is worth noting that an alternative method of synthesis using a classical reaction between calcium oxide and benzoic acid in dmso, affords the same complex in good yield (up to 90%), but does not give X-ray quality crystals. The crystal structure of the title complex, [Ca(benz)_2_(dmso)]_*n*_, is reported herein.

## Structural commentary   

**Figure 1:**
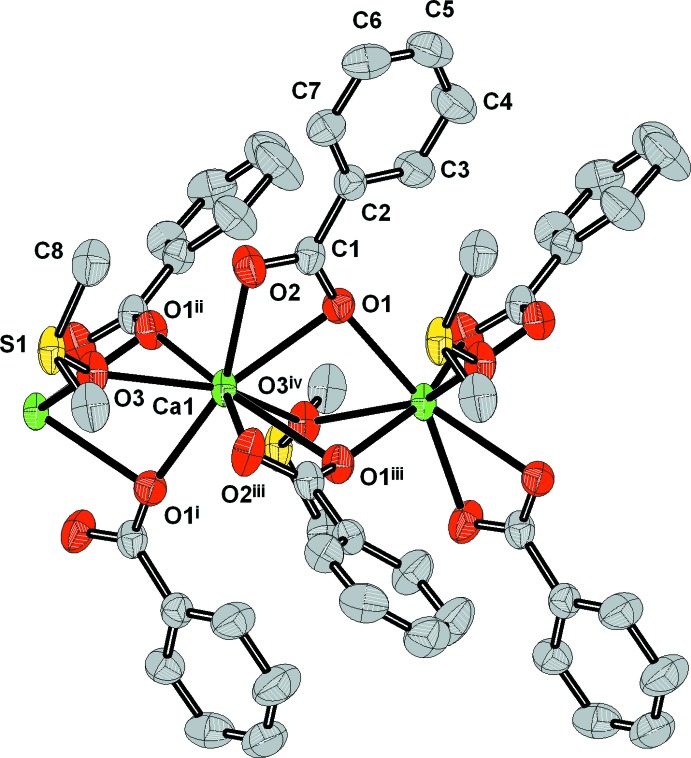
A fragment of the [Ca(benz)_2_(dmso)]_*n*_ chain with the atom-labelling scheme. Displacement ellipsoids are drawn at the 50% probability level. H atoms have been omitted for clarity. For symmetry codes, see Table 1 ▸.

The asymmetric unit of [Ca(benz)_2_(dmso)]_*n*_ comprises one Ca^2+^ cation (site symmetry *m*..), one benzoate ligand and half of a dmso mol­ecule, the other half being generated by mirror symetry. The irregular CaO_8_ coordination polyhedron consists of six O atom donors from two *O,O′* chelating-bridging benzoate carboxyl groups with the same coordination modes, [2.1_1_1_12_] in the Harris notation (Coxall *et al.*, 2000 ▸), and two from μ_2_-bridging dmso mol­ecules (Fig. 1 ▸). The coordination geometry deviates strongly from ideal, the Ca—O bond lengths varying from 2.345 (2) to 2.524 (2) Å (Table 1 ▸) and the O—Ca—O angles from 52.19 (7) to 156.06 (8)°. The bridging Ca1—O1^i^ and Ca1—O1^ii^ (carbox­yl) bond lengths are considerably shorter than the chelate ones, as is usually observed in polymeric benzoates. For the title complex, the bond-valence index [BVS (Ca)] (Allmann, 1975 ▸) is 2.03.

## Supra­molecular features   

**Figure 2:**
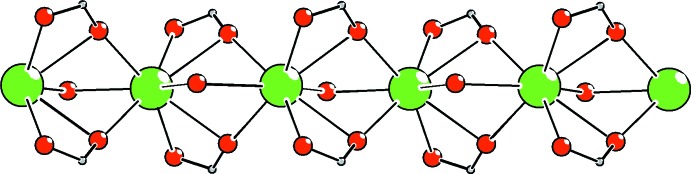
Bridging inter­actions observed in the title complex polymer which extends along the *c-* axis direction. Phenyl rings and H atoms have been omitted for clarity.

**Figure 3:**
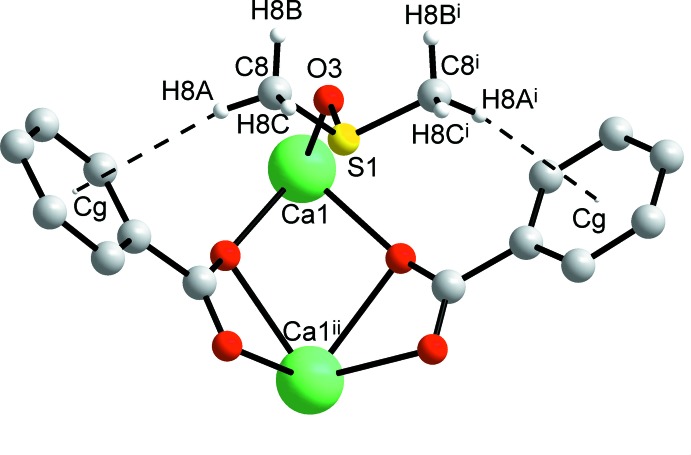
C—H⋯π hydrogen bonds involving a dmso donor as found in the title complex. For symmetry codes, see Table 1 ▸).

The triple-O-bridged CaO_8_ polyhedra form one-dimensional coordination polymeric chains which extend parallel to the *c*-axis direction (Figs. 2 ▸–4 ▸ ▸). The Ca⋯Ca^i^ and Ca1⋯Ca1^iv^ separation in the chain is 3.6401 (5) Å [symmetry code (iv): −*x* + 1, −*y*, *z* − 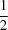]. To the best of our knowledge, this is the first Ca carboxyl­ate polymer based on non-centrosymmetric bridges (μ-η^2^:η^1^)_2_. For bridging modes in coordination polymeric structures, reference should be made to Deacon *et al.* (2007 ▸) and Busskamp *et al.* (2007 ▸). The polymer chains in the title compound are additionally stabilized by weak C—H⋯π inter­actions between the methyl groups of the dmso mol­ecule and the benzoate rings (centroid *Cg*) (Table 2 ▸, Figs. 3 ▸ and 4 ▸).

## Synthesis and crystallization   

Calcium oxide (0.056 g, 1 mmol) and benzoic acid (0.244 g, 2 mmol) were added to 20 ml of dmso and stirred magnetically for *ca* 5 h at 323 K, after which the solution was filtered. The white precipitate which formed after one day was collected and dried in air; yield: 0.4 g (90%). Elemental analysis for C_16_H_16_CaO_5_S (*M*_r_ = 360.43). Calculated: Ca, 11.12%; found: Ca, 11.0%. IR (KBr, cm^−1^): 1603 (*s*), 1562 (*s*), 1405 (*s*), 1024 (*s*), 721 (*s*). Crystals suitable for X-ray analysis were obtained by slow evaporation at room temperature of a solution which was the product from the reaction between manganese powder (0.05 g, 1 mmol), copper powder (0.06 g, 1 mmol), benzohydrazide (0.409 g, 3 mmol), salicyl­aldehyde (0.314 ml, 3 mmol), CaO (0.168 g, 3 mmol), KSCN (0.291 g, 3 mmol) and dmso (20 ml). The reaction was carried out at 353 K with magnetic stirring for eight hours, after which undissolved products were filtered off.

## Refinement details   

Crystal data, data collection and structure refinement details are given in Table 3 ▸. Hydrogen atoms were placed in calculated positions [C—H_aromatic_ = 0.95; C—H_meth­yl_ = 0.99 Å] and were allowed to ride in the refinements, with *U*_iso_(H) = 1.2*U*_eq_(aromatic C) or 1.5*U*_eq_(methyl C). Although not of relevance in this crystal involving achiral mol­ecules, the Flack absolute structure parameter (Flack, 1983 ▸) was determined as 0.04 (8) by classical fit to all intensities and 0.07 (3) from 557 selected quotients (Parsons *et al.*, 2013 ▸).

## Supplementary Material

Crystal structure: contains datablock(s) global, I. DOI: 10.1107/S2056989015012487/zs2334sup1.cif

Structure factors: contains datablock(s) I. DOI: 10.1107/S2056989015012487/zs2334Isup2.hkl

CCDC reference: 1409468

Additional supporting information:  crystallographic information; 3D view; checkCIF report

## Figures and Tables

**Figure 4:**
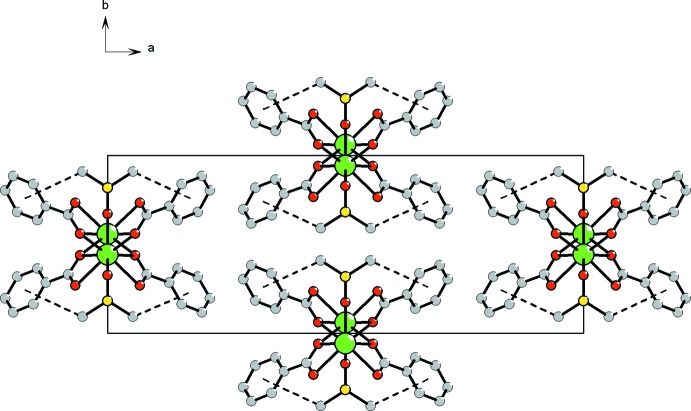
Packing of the mol­ecular chains viewed down the chain direction (the crystallographic *c* axis). C—H⋯π bonds are shown as dashed lines.

**Table 1:** Selected bond lengths ()

Ca1O1^i^	2.345(2)	Ca1O3	2.494(3)
Ca1O1^ii^	2.345(2)	Ca1O3^iv^	2.516(3)
Ca1O2^iii^	2.481(2)	Ca1O1^iii^	2.524(2)
Ca1O2	2.481(2)	Ca1O1	2.524(2)

Symmetry codes: (i) 
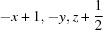
; (ii) 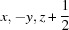; (iii) 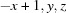; (iv) 
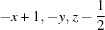
.

**Table 2:** CH interactions (, ) *Cg* is the centroid of the benzoate ring.

*D*H*A*	*D*H	H*A*	*D* *A*	*D*H*A*
C8H8*A* *Cg*	0.96	2.84	3.790(4)	169

**Table 3:** Experimental details

Crystal data	
Chemical formula	[Ca(C_7_H_5_O_2_)_2_(C_2_H_6_OS)]
*M*_r_	360.43
Crystal system, space group	Orthorhombic, *C* *m* *c*2_1_
Temperature (K)	200
*a*, *b*, *c* ()	25.531(2), 9.5351(8), 6.9330(4)
*V* (^3^)	1687.7(2)
*Z*	4
Radiation type	Mo *K*
(mm^1^)	0.52
Crystal size (mm)	0.22 0.15 0.11

Data collection	
Diffractometer	Rigaku Mercury CCD
Absorption correction	Multi-scan (Blessing, 1995 ▸)
*T*_min_, *T*_max_	0.798, 0.951
No. of measured, independent and observed [*I* > 2(*I*)] reflections	3704, 1897, 1676
*R*_int_	0.025
(sin /)_max_ (^1^)	0.683

Refinement	
*R*[*F* ^2^ > 2(*F* ^2^)], *wR*(*F* ^2^), *S*	0.044, 0.103, 1.13
No. of reflections	1897
No. of parameters	109
No. of restraints	1
H-atom treatment	H-atom parameters constrained
_max_, _min_ (e ^3^)	0.59, 0.39
Absolute structure	Flack *x* determined using 557 quotients [(*I* ^+^)(*I* )]/[(*I* ^+^)+(*I* )] (Parsons *et al.*, 2013 ▸)
Absolute structure parameter	0.07(3)

Computer programs: *CrystalClear* (Rigaku, 1999 ▸), *SIR92* (Altomare *et al.*, 1993 ▸)., *SHELXL97* (Sheldrick, 2008 ▸), *DIAMOND* (Brandenburg Putz, 2006 ▸) and *WinGX* (Farrugia, 2012 ▸).

## supplementary crystallographic information

### Crystal data

**Table:**

[Ca(C_7_H_5_O_2_)_2_(C_2_H_6_OS)]	*D*_x_ = 1.418 Mg m^−^^3^
*M**_r_* = 360.43	Mo *K*α radiation, λ = 0.71069 Å
Orthorhombic, *C**m**c*2_1_	Cell parameters from 1872 reflections
*a* = 25.531 (2) Å	θ = 2.3–28.7°
*b* = 9.5351 (8) Å	µ = 0.52 mm^−^^1^
*c* = 6.9330 (4) Å	*T* = 200 K
*V* = 1687.7 (2) Å^3^	Block, colorless
*Z* = 4	0.22 × 0.15 × 0.11 mm
*F*(000) = 752	

### Data collection

**Table:**

Rigaku Mercury CCD (2x2 bin mode) diffractometer	1897 independent reflections
Graphite monochromator	1676 reflections with *I* > 2σ(*I*)
Detector resolution: 14.7059 pixels mm^-1^	*R*_int_ = 0.025
dtprofit.ref scans	θ_max_ = 29.0°, θ_min_ = 2.3°
Absorption correction: multi-scan (Blessing, 1995)	*h* = −34→26
*T*_min_ = 0.798, *T*_max_ = 0.951	*k* = −12→6
3704 measured reflections	*l* = −9→9

### Refinement

**Table:**

Refinement on *F*^2^	Secondary atom site location: difference Fourier map
Least-squares matrix: full	Hydrogen site location: inferred from neighbouring sites
*R*[*F*^2^ > 2σ(*F*^2^)] = 0.044	H-atom parameters constrained
*wR*(*F*^2^) = 0.103	*w* = 1/[σ^2^(*F*_o_^2^) + (0.040*P*)^2^ + 0.7932*P*] where *P* = (*F*_o_^2^ + 2*F*_c_^2^)/3
*S* = 1.13	(Δ/σ)_max_ < 0.001
1897 reflections	Δρ_max_ = 0.59 e Å^−^^3^
109 parameters	Δρ_min_ = −0.39 e Å^−^^3^
1 restraint	Absolute structure: Flack *x* determined using 557 quotients [(*I*^+^)-(*I*^-^)]/[(*I*^+^)+(*I*^-^)] (Parsons *et al.*, 2013)
Primary atom site location: structure-invariant direct methods	Absolute structure parameter: 0.07 (3)

### Special details

**Table:**

Geometry. All e.s.d.'s (except the e.s.d. in the dihedral angle between two l.s. planes) are estimated using the full covariance matrix. The cell e.s.d.'s are taken into account individually in the estimation of e.s.d.'s in distances, angles and torsion angles; correlations between e.s.d.'s in cell parameters are only used when they are defined by crystal symmetry. An approximate (isotropic) treatment of cell e.s.d.'s is used for estimating e.s.d.'s involving l.s. planes.
Refinement. Refinement of *F*^2^ against ALL reflections. The weighted *R*-factor *wR* and goodness of fit *S* are based on *F*^2^, conventional *R*-factors *R* are based on *F*, with *F* set to zero for negative *F*^2^. The threshold expression of *F*^2^ > σ(*F*^2^) is used only for calculating *R*-factors(gt) *etc*. and is not relevant to the choice of reflections for refinement. *R*-factors based on *F*^2^ are statistically about twice as large as those based on *F*, and *R*- factors based on ALL data will be even larger.

### Fractional atomic coordinates and isotropic or equivalent isotropic displacement parameters (Å^2^)

**Table:**

	*x*	*y*	*z*	*U*_iso_*/*U*_eq_	
Ca1	0.5000	0.05825 (7)	0.36175 (11)	0.0325 (2)	
O1	0.44205 (8)	0.06089 (19)	0.0668 (3)	0.0388 (5)	
O2	0.43213 (9)	0.2334 (2)	0.2748 (4)	0.0509 (6)	
O3	0.5000	0.1724 (3)	0.6855 (5)	0.0414 (7)	
C1	0.41769 (12)	0.1682 (3)	0.1275 (5)	0.0375 (7)	
C2	0.37045 (12)	0.2146 (3)	0.0171 (5)	0.0388 (7)	
C3	0.35274 (13)	0.1367 (4)	−0.1381 (7)	0.0589 (10)	
H3	0.3695	0.0533	−0.1704	0.071*	
C4	0.31036 (16)	0.1818 (5)	−0.2452 (8)	0.0756 (13)	
H4	0.2988	0.1289	−0.3495	0.091*	
C5	0.28529 (14)	0.3044 (4)	−0.1984 (7)	0.0624 (11)	
H5	0.2567	0.3340	−0.2707	0.075*	
C6	0.30222 (15)	0.3831 (4)	−0.0461 (7)	0.0584 (11)	
H6	0.2855	0.4669	−0.0160	0.070*	
C7	0.34453 (12)	0.3375 (3)	0.0639 (6)	0.0464 (8)	
H7	0.3555	0.3900	0.1695	0.056*	
C8	0.44725 (16)	0.4090 (3)	0.6588 (6)	0.0560 (10)	
H8A	0.4146	0.3721	0.7051	0.084*	
H8B	0.4493	0.3964	0.5217	0.084*	
H8C	0.4494	0.5071	0.6889	0.084*	
S1	0.5000	0.31863 (10)	0.77164 (16)	0.0444 (3)	

### Atomic displacement parameters (Å^2^)

**Table:**

	*U*^11^	*U*^22^	*U*^33^	*U*^12^	*U*^13^	*U*^23^
Ca1	0.0504 (4)	0.0257 (3)	0.0214 (4)	0.000	0.000	0.0005 (3)
O1	0.0472 (11)	0.0343 (11)	0.0349 (13)	0.0085 (8)	0.0000 (10)	−0.0033 (9)
O2	0.0720 (14)	0.0427 (12)	0.0381 (13)	0.0142 (11)	−0.0139 (13)	−0.0064 (11)
O3	0.069 (2)	0.0218 (12)	0.0333 (17)	0.000	0.000	−0.0020 (12)
C1	0.0515 (17)	0.0302 (14)	0.0307 (17)	0.0017 (13)	0.0019 (13)	0.0022 (12)
C2	0.0421 (15)	0.0389 (16)	0.0353 (17)	0.0002 (13)	0.0011 (13)	0.0042 (14)
C3	0.0556 (18)	0.060 (2)	0.061 (2)	0.0180 (16)	−0.014 (2)	−0.022 (2)
C4	0.067 (2)	0.086 (3)	0.074 (3)	0.020 (2)	−0.031 (2)	−0.022 (3)
C5	0.0483 (19)	0.069 (2)	0.070 (3)	0.0101 (19)	−0.0107 (18)	0.010 (2)
C6	0.0478 (19)	0.045 (2)	0.082 (3)	0.0097 (16)	0.0054 (19)	0.003 (2)
C7	0.0483 (17)	0.0390 (17)	0.052 (2)	0.0063 (14)	0.0025 (16)	−0.0028 (16)
C8	0.083 (3)	0.0357 (15)	0.049 (2)	0.0096 (17)	0.005 (2)	−0.0010 (17)
S1	0.0829 (8)	0.0257 (5)	0.0244 (6)	0.000	0.000	−0.0015 (4)

### Geometric parameters (Å, º)

**Table:**

Ca1—O1^i^	2.345 (2)	C1—C2	1.496 (4)
Ca1—O1^ii^	2.345 (2)	C2—C3	1.383 (5)
Ca1—O2^iii^	2.481 (2)	C2—C7	1.384 (4)
Ca1—O2	2.481 (2)	C3—C4	1.381 (5)
Ca1—O3	2.494 (3)	C3—H3	0.9300
Ca1—O3^iv^	2.516 (3)	C4—C5	1.371 (5)
Ca1—O1^iii^	2.524 (2)	C4—H4	0.9300
Ca1—O1	2.524 (2)	C5—C6	1.365 (6)
Ca1—C1^iii^	2.855 (3)	C5—H5	0.9300
Ca1—C1	2.855 (3)	C6—C7	1.392 (5)
Ca1—Ca1^i^	3.6401 (5)	C6—H6	0.9300
Ca1—Ca1^iv^	3.6401 (5)	C7—H7	0.9300
O1—C1	1.269 (3)	C8—S1	1.780 (4)
O1—Ca1^iv^	2.345 (2)	C8—H8A	0.9600
O2—C1	1.251 (4)	C8—H8B	0.9600
O3—S1	1.517 (3)	C8—H8C	0.9600
O3—Ca1^i^	2.516 (3)	S1—C8^iii^	1.780 (4)
			
O1^i^—Ca1—O1^ii^	78.22 (11)	C1^iii^—Ca1—Ca1^i^	130.82 (7)
O1^i^—Ca1—O2^iii^	91.88 (8)	C1—Ca1—Ca1^i^	130.82 (7)
O1^ii^—Ca1—O2^iii^	156.06 (8)	O1^i^—Ca1—Ca1^iv^	115.44 (6)
O1^i^—Ca1—O2	156.06 (8)	O1^ii^—Ca1—Ca1^iv^	115.44 (6)
O1^ii^—Ca1—O2	91.88 (8)	O2^iii^—Ca1—Ca1^iv^	88.51 (6)
O2^iii^—Ca1—O2	88.60 (12)	O2—Ca1—Ca1^iv^	88.51 (6)
O1^i^—Ca1—O3	70.48 (7)	O3—Ca1—Ca1^iv^	171.90 (7)
O1^ii^—Ca1—O3	70.48 (7)	O3^iv^—Ca1—Ca1^iv^	43.17 (8)
O2^iii^—Ca1—O3	85.70 (8)	O1^iii^—Ca1—Ca1^iv^	39.79 (5)
O2—Ca1—O3	85.70 (8)	O1—Ca1—Ca1^iv^	39.79 (5)
O1^i^—Ca1—O3^iv^	82.59 (8)	C1^iii^—Ca1—Ca1^iv^	64.56 (7)
O1^ii^—Ca1—O3^iv^	82.59 (8)	C1—Ca1—Ca1^iv^	64.56 (7)
O2^iii^—Ca1—O3^iv^	118.06 (7)	Ca1^i^—Ca1—Ca1^iv^	144.47 (4)
O2—Ca1—O3^iv^	118.06 (7)	C1—O1—Ca1^iv^	154.2 (2)
O3—Ca1—O3^iv^	144.93 (11)	C1—O1—Ca1	91.54 (19)
O1^i^—Ca1—O1^iii^	97.25 (7)	Ca1^iv^—O1—Ca1	96.69 (7)
O1^ii^—Ca1—O1^iii^	149.97 (5)	C1—O2—Ca1	94.01 (18)
O2^iii^—Ca1—O1^iii^	52.19 (7)	S1—O3—Ca1	139.05 (18)
O2—Ca1—O1^iii^	101.88 (8)	S1—O3—Ca1^i^	127.75 (19)
O3—Ca1—O1^iii^	136.43 (6)	Ca1—O3—Ca1^i^	93.19 (9)
O3^iv^—Ca1—O1^iii^	67.37 (7)	O2—C1—O1	121.8 (3)
O1^i^—Ca1—O1	149.97 (5)	O2—C1—C2	120.6 (3)
O1^ii^—Ca1—O1	97.25 (7)	O1—C1—C2	117.6 (3)
O2^iii^—Ca1—O1	101.88 (8)	O2—C1—Ca1	60.08 (16)
O2—Ca1—O1	52.19 (7)	O1—C1—Ca1	62.08 (16)
O3—Ca1—O1	136.43 (6)	C2—C1—Ca1	173.4 (2)
O3^iv^—Ca1—O1	67.37 (7)	C3—C2—C7	118.8 (3)
O1^iii^—Ca1—O1	71.78 (10)	C3—C2—C1	120.2 (3)
O1^i^—Ca1—C1^iii^	93.35 (8)	C7—C2—C1	121.1 (3)
O1^ii^—Ca1—C1^iii^	170.72 (8)	C4—C3—C2	120.4 (3)
O2^iii^—Ca1—C1^iii^	25.91 (8)	C4—C3—H3	119.8
O2—Ca1—C1^iii^	97.39 (9)	C2—C3—H3	119.8
O3—Ca1—C1^iii^	110.58 (8)	C5—C4—C3	120.3 (4)
O3^iv^—Ca1—C1^iii^	92.57 (8)	C5—C4—H4	119.9
O1^iii^—Ca1—C1^iii^	26.38 (7)	C3—C4—H4	119.9
O1—Ca1—C1^iii^	88.11 (8)	C6—C5—C4	120.2 (4)
O1^i^—Ca1—C1	170.72 (8)	C6—C5—H5	119.9
O1^ii^—Ca1—C1	93.35 (8)	C4—C5—H5	119.9
O2^iii^—Ca1—C1	97.39 (9)	C5—C6—C7	119.9 (3)
O2—Ca1—C1	25.91 (8)	C5—C6—H6	120.1
O3—Ca1—C1	110.58 (8)	C7—C6—H6	120.1
O3^iv^—Ca1—C1	92.57 (8)	C2—C7—C6	120.4 (3)
O1^iii^—Ca1—C1	88.11 (8)	C2—C7—H7	119.8
O1—Ca1—C1	26.38 (7)	C6—C7—H7	119.8
C1^iii^—Ca1—C1	94.77 (13)	S1—C8—H8A	109.5
O1^i^—Ca1—Ca1^i^	43.52 (5)	S1—C8—H8B	109.5
O1^ii^—Ca1—Ca1^i^	43.52 (5)	H8A—C8—H8B	109.5
O2^iii^—Ca1—Ca1^i^	115.90 (7)	S1—C8—H8C	109.5
O2—Ca1—Ca1^i^	115.90 (7)	H8A—C8—H8C	109.5
O3—Ca1—Ca1^i^	43.63 (7)	H8B—C8—H8C	109.5
O3^iv^—Ca1—Ca1^i^	101.29 (8)	O3—S1—C8^iii^	105.78 (14)
O1^iii^—Ca1—Ca1^i^	140.76 (5)	O3—S1—C8	105.78 (14)
O1—Ca1—Ca1^i^	140.76 (5)	C8^iii^—S1—C8	98.4 (3)

Symmetry codes: (i) −*x*+1, −*y*, *z*+1/2; (ii) *x*, −*y*, *z*+1/2; (iii) −*x*+1, *y*, *z*; (iv) −*x*+1, −*y*, *z*−1/2.

### Hydrogen-bond geometry (Å, º)

Cg is the centroid of the benzoate ring.

**Table:**

*D*—H···*A*	*D*—H	H···*A*	*D*···*A*	*D*—H···*A*
C8—H8*A*···*Cg*	0.96	2.84	3.790 (4)	169
